# A dosimetry study of post‐mastectomy radiation therapy with AeroForm tissue expander

**DOI:** 10.1002/acm2.12962

**Published:** 2020-07-02

**Authors:** Seng Boh Lim, Li Cheng Kuo, Guang Li, Hsiang‐Chi Kuo, Beryl McCormick, Oren Cahlon, Simon Powell, Linda X. Hong

**Affiliations:** ^1^ Department of Medical Physics Memorial Sloan Kettering Cancer Center New York NY USA; ^2^ Department of Radiation Oncology Memorial Sloan Kettering Cancer Center New York NY USA

**Keywords:** breast cancer, post‐mastectomy radiation therapy, AeroForm tissue expander, film dosimetry, OLSD dosimetry

## Abstract

**Purpose:**

To evaluate the dosimetric effects of the AeroForm^TM^ (AirXanpders®, Palo Alto, CA) tissue expander in‐situ for breast cancer patients receiving post‐mastectomy radiation therapy.

**Methods and Materials:**

A film phantom (P1) was constructed by placing the metallic canister of the AeroForm on a solid water phantom with EBT3 films at five depths ranging from 2.6 mm to 66.2 mm. A breast phantom (P2), a three‐dimensional printed tissue‐equivalent breast with fully expanded AeroForm in‐situ, was placed on a thorax phantom. A total of 21 optical luminescent dosimeters (OLSDs) were placed on the anterior skin–gas interface and the posterior chest wall–metal interface of the AeroForm. Both phantoms were imaged with a 16‐bit computed tomography scanner with orthopedic metal artifact reduction. P1 was irradiated with an open field utilizing 6 MV and 15 MV photon beams at 0°, 90°, and 270°. P2 was irradiated using a volumetric modulated arc therapy plan with a 6 MV photon beam and a tangential plan with a 15 MV photon beam. All doses were calculated using Eclipse (Varian, Palo Alto, CA) with AAA and AcurosXB (AXB) algorithms.

**Results:**

The average dose differences between film measurements and AXB in the region adjacent to the canister in P1 were within 3.1% for 15 MV and 0.9% for 6 MV. Local dose differences over 10% were also observed. In the chest wall region of P2, the median dose of OLSDs in percentage of prescription dose were 108.4% (range 95.4%–113.0%) for the 15MV tangential plan and 110.4% (range 99.1%–113.8%) for the 6MV volumetric modulated arc therapy plan. In the skin–gas interface, the median dose of the OLSDs were 102.3% (range 92.7%–107.7%) for the 15 MV plan and 108.2% (range 97.8–113.5%) for the 6 MV plan. Measured doses were, in general, higher than calculated doses with AXB calculations. The AAA dose algorithms produced results with slightly larger discrepancies between measurements compared with AXB.

**Conclusions:**

The AeroForm creates significant dose uncertainties in the chest wall–metal interface. The AcurosXB dose calculation algorithm is recommended for more accurate calculations. If possible, post‐mastectomy radiation therapy should be delivered after the permanent implant is in place.

## 1 INTRODUCTION

Post‐mastectomy radiation therapy (PMRT) to the chest wall and locoregional nodes has been shown to reduce the incidence of locoregional and distant recurrence and to improve overall survival in patients with node‐positive breast cancer.^1^, ^2^, ^3^ At our institution, we have a well‐established planning protocol for patients with McGhan style tissue expander in situ. If the chest wall is irradiated with tangential beams, 15 MV (15X) photon beams are used for better transmission through the expander’s metal (compared with 6 MV photon tangents) and 1cm bolus is used to provide adequate skin dose.^4^ If Volumetric Modulated Arc Therapy (VMAT) techniques is used, 6 MV (6X) photon arcs with a 0.3 cm bolus are used.^5^

A new tissue expander system, the AeroForm™ (AirXpanders®, Palo Alto, CA), has recently become available to patients.^6^, ^7^, ^8^, ^9^ Aeroform expander consists of an implantable silicone tissue expander containing a metal reservoir of compressed carbon dioxide. The metallic reservoir causes a significant attenuation and inhomogeneous doses around the expander in PMRT^9^, ^10^, ^11^, ^12^ with ^60^Co and 6MV. Moni^10^ has shown that the treatment planning system (TPS) can have significant dose error (>5%) at the chest wall–expander interface. The computed tomography (CT) high‐Z artifact associated with the device creates additional dosimetric challenges. Shah^13^ suggests that further studies on the potential increase in toxicity, underdosing, and tissue delineation challenges associated with this gas expander in situ during radiotherapy is warranted.

In this study, we investigate the feasibility of our current clinical planning practice for patients with tissue expander in situ which was not addressed in published studies. We focus on the dosimetry of the AeroForm in the chest wall–metal and skin–gas interfaces to develop treatment planning guidelines for patients who are receiving PMRT with the AeroForm expander. The dosimetry of skin–gas interface of the Aeroform, which would have impacted on the skin dose of the patient, has also not been addressed by other studies. The impact of different assignment of Hounsfield units (HU) and materials of the Aeroform on 16‐bit CT scans was also investigated.

## 2 METHODS AND MATERIALS

### 2.1 Film phantom (P1) and breast phantom (P2)

**Fig. 1:**
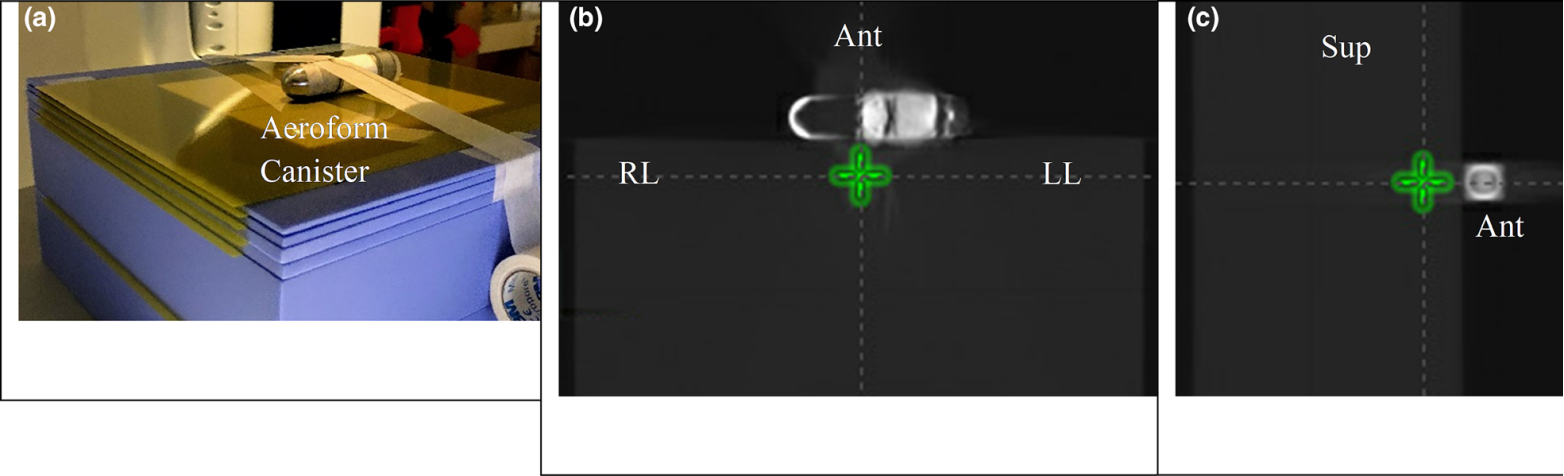
Computed tomography images for the film phantom P1. (a) P1 setup with canister; (b) Transverse view of canister on solid water (25 cm × 25 cm × 12 cm). c) Sagittal view of canister on solid water. Green marker indicates isocenter location for calculations and measurements. ^*^anterior (Ant), ^†^left lateral (LL), ^‡^right lateral (RL), ^§^superior (Sup)

For film dosimetry, the metallic canister was removed from an AeroForm expander and placed on top of a 25 × 25 × 12 cm Solid Water^®^ phantom (Gammex, Melbourne, FL). Gafchromic films (Ashland, Bridgewater, NJ) were inserted at 0.2, 0.5, 1.0, 1.5, and 6 cm in the phantom (P1, Fig. 1).

**Fig. 2:**
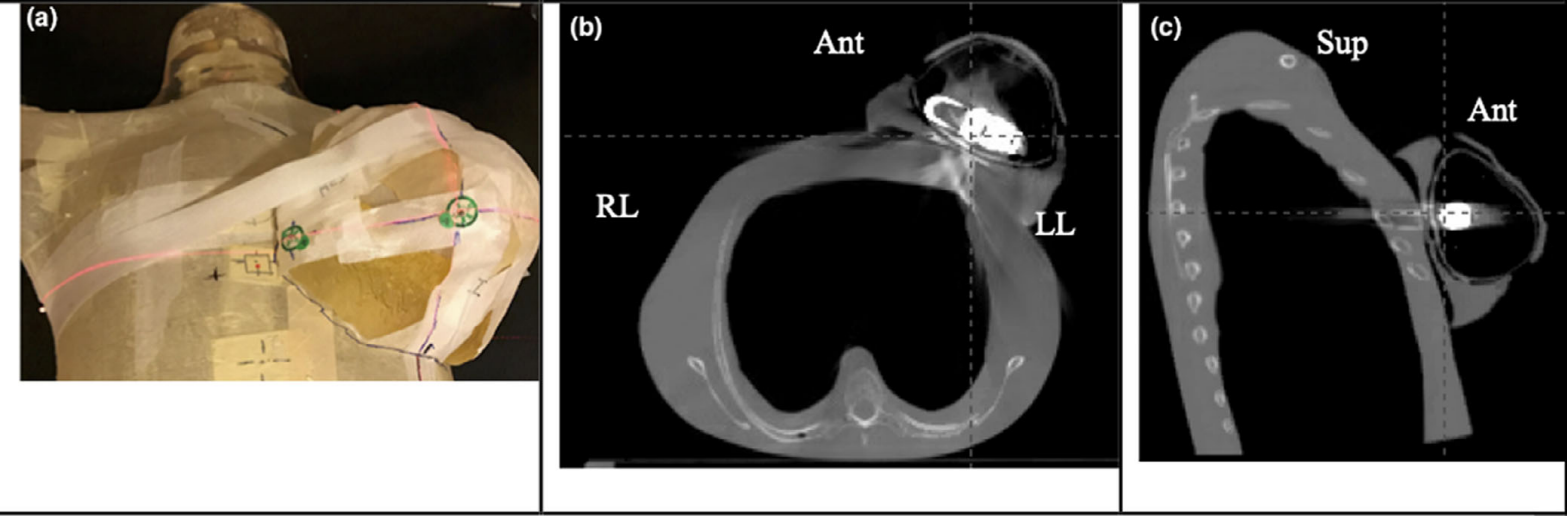
The breast phantom P2. (a) 3D printed tissue‐equivalent breast with in situ expander taped on a plastic phantom. (b) Transverse view of P2. The anterior (Ant) part of the 3D breast. (c) Sagittal view of P2. ^*^left lateral (LL), ^†^right lateral (RL), ^‡^superior (Sup)

An intact AeroForm expander was inflated to full capacity (400 cm^3^). Three‐dimensional (3D) printing technology was used to create a breast phantom with TangoPlus FLX930 tissue‐equivalent material (Stratasys, Eden Prairie, MN) with the expander in situ. The 3D printed "breast” was taped to a plastic phantom as the breast phantom (P2) to simulate a post‐mastectomy patient (Fig. 2). The anterior part of the 3D breast in P2 is 0.5 cm thick, mimicking the patient’s skin.

Multiple optically stimulated luminescence dosimeters (OSLD) (LANDAUER, Glenwood IL) were placed throughout this 3D printed breast to measure doses in the chest wall–metal and skin–gas interfaces. In all, 12 OLSDs were used to generate a calibration curve from 100 to 700 cGy. Another 50 OSLDs were used in the clinical study. To improve the dosimetric accuracy, each OSLD, *i*, was pre‐irradiated with a calibration dose, D_c_, of 200cGy and was read out as D_c,i_. Chip‐specific sensitivity factor, SF_i,_, was obtained as follows:

$$SF_{i}=\frac{D_{c,i}}{D_{c}}$$

A total of eight chips were taken from the clinical chips as reference chips and were grouped into two equal groups. Each group was irradiated with the prescribed energy and dose, D_p_, of the clinical plan. The average of the net dose, defined by subtracting D_c,i_ from the read‐out, deposited on each chip, D_p,i_, was used to generate the correction factor, CF, as follows:

$$CF=\frac{\left\langle SF_{i}D_{p,i}\right\rangle }{D_{p}}$$

The clinical dose of each OSLD, D_i_, was determined by correcting the net clinical dose, D_o,i_, as:

$$D_{i}=\left(D_{o,i}SF_{i}\right)CF.$$

**Fig. 3:**
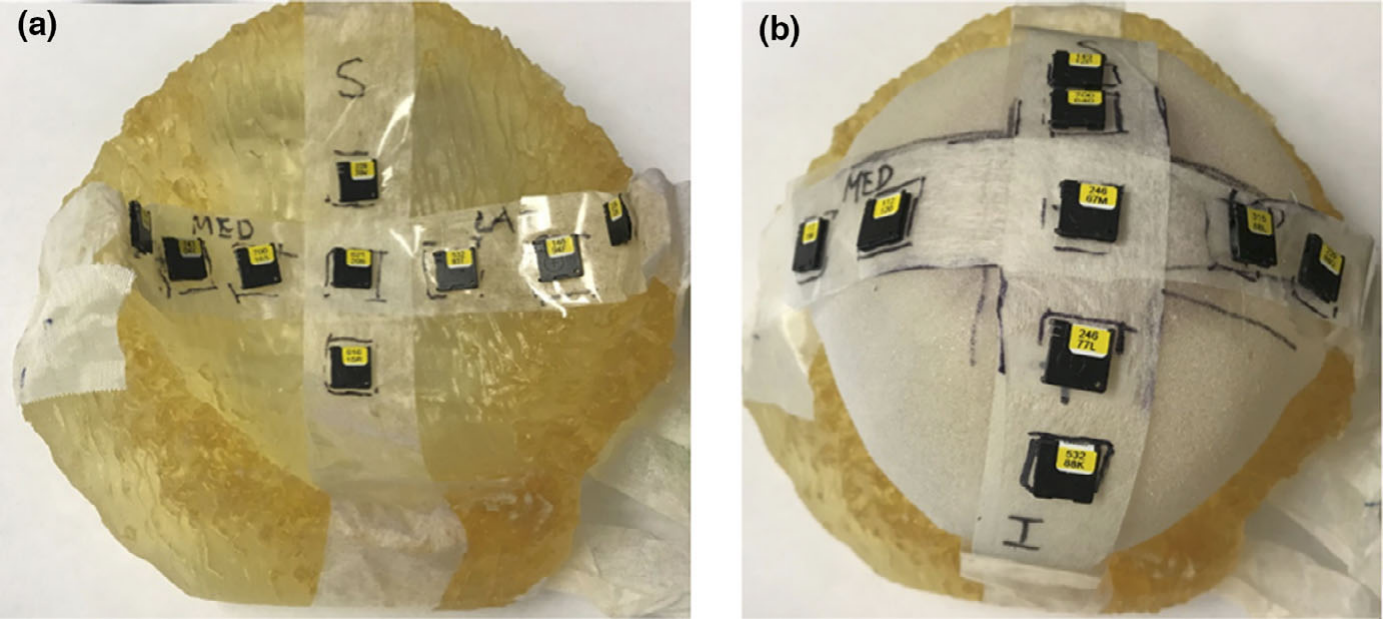
OLSD placement throughout the P2 phantom. (a) OLSD placement in the chest wall–AeroForm interface in the 3D printed breast posterior of the AeroForm. (b) OLSD placement in the AeroForm–Skin interface without 3D printed skin

The clinical OSLDs were placed on the interfaces of the breast phantom. A set of nine OLSDs were placed in the chest wall–AeroForm (Fig. 3a) and AeroForm–skin interfaces underneath the 0.5 cm 3D printed skin (Fig. 3b) in P2.

### 2.2 Treatment planning

Both P1 and P2 were scanned with 2.0 mm slice in Brilliance BigBore CT scanner (Philips, Amsterdam, Netherlands) with 16 bits and orthopedic metal artifact reduction (OMAR).^14^, ^15^ Treatment planning for both phantoms was completed with Eclipse V13.6 (Varian, Palo Alto, CA). For P1, 6X and 15X 10 × 10 cm open fields in the anterior and lateral directions were calculated with the beam’s isocenter 1.5 cm deep, as shown in Figure 1. Four hundred cGy was delivered at each beam to provide sufficient signals to the films.

For P2, two different treatment techniques, with the prescription of 200 cGy and 265 cGy for 6x and 15x corresponding to the typical dose‐per‐fraction that are currently used clinically for the saline‐filled expander in our institution were investigated: (1) tangential fields with 15X and 1 cm bolus^4^ (C1) and (2) VMAT plan with four partial arcs, 6X, and 0.3 cm bolus^5^ (C2). Both plans were planned according to our clinical protocols^4^, ^5^ and procedures.^16^ The canister, air inside the AeroForm, the artifacts in the soft tissue area, and all the OSLDs were contoured.

All plans were calculated with both AAA and AXB algorithms. The plans were generated with the 16‐bit CT. A 16‐bit Hounsfield unit (HU) electron density conversion curve was generated using Gammex CT electron density phantom with additional titanium and stainless steel (Gammex, Melbourne, FL). The impact of CT number assignments with AAA and AXB dose algorithms was investigated. The plans without re‐assignments were labeled as AAA‐16 bits with AAA and AXB‐16 bits with AXB calculations. The plans with HU re‐assignment to the canister (titanium alloy, HU = 4800), air inside the AeroForm (Air, HU = −1000), and artifacts in soft tissue (tissue, HU = 100) were recalculated and labeled as AAA‐4800 and AXB‐4800. To simulate the HU saturation in 12‐bit CT, the pixel with HU greater than 3071 was re‐assigned to 3071 and were labeled as AAA‐12 bit and AXB‐12 bit for AAA and AXB calculations.

### 2.3 Measurements and analysis

All measurements were delivered on a Varian TrueBeam with M120 MLC. The three‐channel calibration protocol,^17^ using FilmQA Pro (Ashland, Bridgewater, NJ), was used to calibrate the films. In‐house software was used to register the calibrated films to the TPS calculation. A rectangular (10 × 2 cm) region of interest (ROI) was used to determine the average dose difference, <ΔD_ROI_>, at each film plane.

The maximum (max), minimum (min), mean dose (<ΔD>), and standard deviation (σ) of the point dose difference between OSLD measurements and calculations were calculated for each CT and algorithm. The significance of the <ΔD> was assessed with a paired mean t‐test.^18^ CBCTs were used to setup the breast phantom before delivery of each plan. OLSDs were contoured on the CBCT images and fused with simulation CTs to reduce dosimetric uncertainty.

## 3 RESULTS

**Fig. 4:**
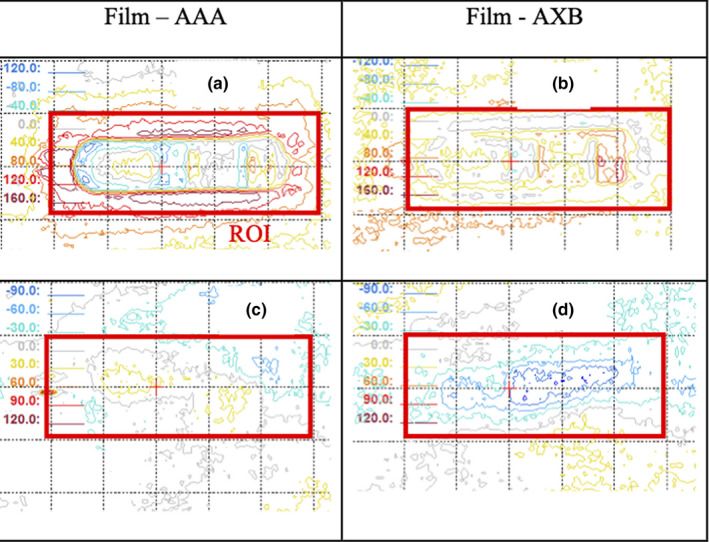
Film dosimetry analysis of P1 at 2.6 mm behind the AeroForm canister with anterior–posterior open field; (a) dose difference between film and AAA for 6X; (b) dose difference between film and AXB‐4800 for 6X; (c) dose difference film and AAA for 15X; (d) dose difference between film and AXB‐4800 for 15X

**Fig. 5:**
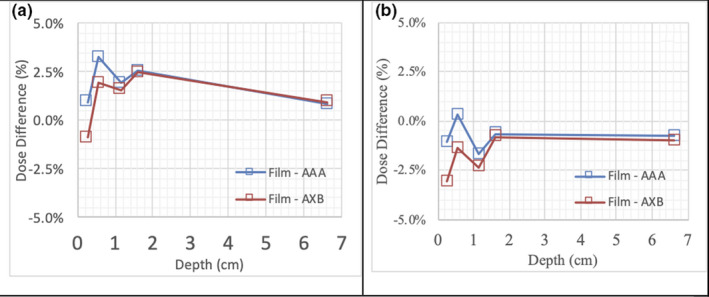
Dose difference between film measurements and AAA and AXB‐4800 calculation algorithms: (a) 6X anterior and bilateral beams setup; (b) 15X bilateral beams setup

Figure 4 illustrates the film results. At 2.6 mm downstream from the canister, with a 6X anterior–posterior (AP) open field, the average dose differences within the ROI, <ΔD_ROI_> for AAA and AXB were 1.4% and 3.8%, respectively. Local dose differences amounting to ±14% and ± 8% for AAA and AXB, respectively, were observed for 6X. The <ΔD_ROI_> was within ± 3.4% for both energies and algorithms. In a composite beam arrangement of AP plus bilateral fields mimicking C2, the <ΔD_ROI_> was 3.9% and 2.0% for AAA and AXB, respectively (Fig. 4b and c). The local dose errors were within 10% for AAA and 6% for AXB. The <ΔD_ROI_> of the AP open field for 15X were −1.1% and −3.1% for AAA and AXB, respectively. However, the local errors were over 10% for both algorithms. The analysis also revealed significant dose variation from films behind the region of the canister comprising high Z materials. In the case of 15X, over 20% local differences were observed for both algorithms. A pair of bilateral tangential fields, modeling C1, were measured and had a <ΔD_ROI_> of −0.6 and −2.6 for AAA and AXB at the same depth (Fig. 4d and e). The local dose errors were 2% and 4% for AAA and AXB, respectively. The agreement between measurements and calculation improved with increasing distance from the device for all single beam arrangements. By 16 mm, the dose difference was within 3.4% for both energies and algorithms. The lateral beam arrangements from both energies showed significantly less local variation than the AP setup. The local dose differences were within ±4% for both energies. The <ΔD_ROI_> of C2 in the chest wall region had a variation within 3.2% and 2.5% for AAA and AXB, respectively, from 26 mm to 66.2 mm in depth (Fig. 5a). Although both algorithms showed similar <ΔD_ROI_>, AAA showed more local variation than AXB. The <ΔD_ROI_> of C1 were within 1.4% and 2.6% for AAA and AXB, respectively (Fig. 5b).

The <SF_i_> was found to be 0.967 (σ = 2.9%). The CF for 6x and 15x were found to be 0.98 and 0.97, respectively. Table 1 shows the summary of the dose differences between OLSD and dose calculations for P2. In the chest wall region, the AAA showed a 3.2%–5.9% higher <ΔD>. Statistically insignificant <ΔD> for AXB was found. In the AeroForm–skin interface, both algorithms showed statistically insignificant <ΔD>. In terms of local dose difference, AAA and AXB showed a variation of 16.6% and 10.6%, respectively. In terms of skin dose, both algorithms showed statistically insignificant <ΔD>. Re‐assigning HU of the CT image with both 4800 and 3071 also did not show any significant dosimetric impact.

**Table 1:** Summary of dose difference between optical luminescent dosimeters and calculations with AAA and AXB algorithms using two different energies: 6 MV (6X) and 15 MV (15X) for a breast phantom (P2). P2 was imaged with 16‐bit CT*. The high Z materials of the CT were overridden with 4800 HU and 3071 to provide accurate electron density and mimic 12‐bit CT, respectively

	6x					15x				
	AAA 16bit	AAA 4800	AAA 12bit	AXB‐4800	AXB 12bit	AAA 16bit	AAA 4800	AAA 12bit	AXB 4800	AXB 12bit
Chest wall										
min	−0.9%	−2.2%	−5.1%	−2.9%	−5.3%	−4.4%	−5.8%	−8.1%	−5.9%	−7.4%
max	16.6%	13.8%	11.1%	11.0%	10.1%	9.6%	9.9%	9.5%	4.9%	7.4%
<ΔD>^†^	5.9%	4.6%	3.2%	1.6%	0.8%	4.2%	3.9%	3.2%	0.4%	2.0%
σ	5.8%	5.2%	5.2%	4.2%	4.3%	4.8%	5.3%	6.0%	3.1%	4.8%
*P* value	0.01	0.03	0.11	0.41	0.68	0.05	0.07	0.13	0.81	0.31
Air cavity										
min	−3.3%	−2.5%	−2.6%	−4.4%	−3.7%	−10.8%	−9.7%	−9.8%	−11.6%	−10.8%
max	5.1%	6.0%	5.9%	7.1%	7.0%	4.6%	5.5%	5.4%	3.8%	4.5%
<ΔD>	1.0%	1.6%	1.3%	0.9%	1.7%	−0.9%	−0.6%	−0.8%	−2.7%	−1.3%
σ	3.1%	3.3%	3.4%	4.1%	4.2%	4.3%	4.1%	4.1%	4.2%	4.1%
*P* value	0.68	0.50	0.55	0.70	0.49	0.6	0.7	0.6	0.1	0.4
Skin										
<ΔD>	−4.1%	−3.9%	−3.9%	−5.0%	−2.5%	0.0%	0.2%	0.2%	−1.6%	0.1%
*P* value	0.35	0.40	0.40	0.22	0.5	0.98	0.93	0.94	0.45	1.0

* CT: computed tomography.

^†^ <ΔD>: mean dose.

For the P2 phantom with the 15X tangential plan, the median doses of the OLSDs (in percentage of the prescription dose) were 108.4% (range 95.4%–113.0%) in the chest wall region and 102.3% (range 92.7%–107.7%) in the skin–gas interface. For the P2 phantom with the 6X VMAT plan, the median doses of the OLSDs were 110.4% (range 99.1%–113.8%) in the chest wall region and 108.2% (range 97.8%–113.5%) in the skin–gas interface. These data indicated that both plans delivered adequate clinical doses compared with the prescription dose.

## 4 DISCUSSION

The investigation showed there were no statistically significant <ΔD> between 12‐bit and 16‐bit CT. However, significant artifacts were present on CT images of the AeroForm expander, even with the 16‐bit CT with OMAR. For facilities without 16‐bit CT and OMAR, the artifacts caused by the AeroForm will be more pronounced resulting in difficulty of delineating the normal structures such as chest wall and heart. With 12‐bit CT, the high Z materials saturate at a lower HU which requires manual assignment to improve dose calculation accuracy.^10^, ^11^, ^12^ In addition, we found the limited 12‐bit dynamic range also increased TPS calculation uncertainties.

Although the <ΔD_ROI_> were modest, significant local dosimetric differences (over 20%) were found in P1 phantoms within 5 mm of the canister interfaces. This can be attributed to the inaccurate forward scatter modeling of the high Z materials by both algorithms. However, this study has shown the local dosimetric errors can be moderated with the current clinical beam arrangement.^5^, ^16^

The results from P2, approximately correspond to the results at 5 mm in P1, showed that AAA has higher dosimetric uncertainty than AXB. Significant dose uncertainty (~16.6% locally) at chest wall–metal interface can result in potential underestimation of heart dose. This is perhaps partly attributed to the inaccurate forward and back scatter of the dose calculation algorithms near high Z materials. On the skin and the skin–gas interface, both algorithms showed reasonable dose agreement with measurements (5%–7%). These uncertainties should be incorporated into the clinical planning process.

## CONFLICT OF INTEREST

All the authors have no conflicts of interest.
